# REVISITING THE TEACHING NURSING HOME: IMPACT ON NURSING STUDENTS’ PERCEPTIONS OF WORKING IN LONG-TERM CARE

**DOI:** 10.1093/geroni/igac059.3071

**Published:** 2022-12-20

**Authors:** Howard Degenholtz, Marie Boltz, Donna Fick, Nancy Hodgson, Kim Strauch, Jacob Kariuki, Yurun Cai, Dawn Pajerski

**Affiliations:** University of Pittsburgh, Pittsburgh, Pennsylvania, United States; Penn State, State College, Pennsylvania, United States; Penn State University, University Park, Pennsylvania, United States; University of Pennsylvania, Philadelphia, Pennsylvania, United States; Penn School of Nursing, Philadelphia, Pennsylvania, United States; University of Pittsburgh School of Nursing, Pittsburgh, Pennsylvania, United States; University of Pittsburgh, Pittsburgh, Pennsylvania, United States; University of Pittsburgh School of Nursing, Pittsburgh, Pennsylvania, United States

## Abstract

Revisiting the Teaching Nursing Home is a two-year pilot project to address the long-term care workforce shortage by introducing nursing students to geriatric nursing while improving quality of care within nursing homes. The initiative has multiple components: enhanced clinical rotations for nursing students with partner schools of nursing, implementation of the Institute for Healthcare Improvement Age-Friendly Health System “4M” quality improvement model, and an online learning network. Nursing students at three schools of nursing participated in the clinical rotations at regional nursing homes. The experience was limited to students in one specific course at each school of nursing. At the beginning and end of the spring 2022 semester, students rated their competence in: patient assessment, collaborating with the care team, gathering clinical information, medication review, eliciting resident values, and health promotion. Students also rated their preferences for working in long-term care and with older adults. Data from 85 responses at the start of semester and 64 responses to the end of semester survey were analyzed. Analysis of student responses found that students self-rated competencies improved in all areas except eliciting resident values. Prior to their clinical experience, students ranked working in long-term care and with older adults lower than other settings or populations. The rankings were unchanged after their clinical experiences. These initial findings suggest that although the Teaching Nursing Home Program is meeting the pedagogical goals, attitudinal shifts may require different strategies.

